# Experimental protocols used in tactile perception of materials: a mini review

**DOI:** 10.3389/fpsyg.2026.1926895

**Published:** 2026-10-16

**Authors:** Simran A. Sutavane, Subhash H. Risbud

**Affiliations:** Department of Materials Science Engineering, University of California, Davis, Davis, CA, United States

**Keywords:** haptics, materials, sensory perception, tactile perception, touch

## Abstract

Tactile perception plays a central role in how humans evaluate and interact with materials, influencing perceived roughness, softness, friction, warmth, wetness, comfort, quality, and usability. In recent years, experimental studies on tactile perception have expanded across diverse material systems, including textiles, synthetic fibers, plastics, wood, building materials, soft rubbers, textured surfaces, and haptic overlays. However, the experimental protocols used across these studies remain highly varied. This mini-review examines recent experimental approaches adopted in tactile perception studies of materials, with emphasis on work published within approximately the last 5 years. We identified common methodological strategies, including psychophysical rating scales, forced-choice and pairwise-comparison tasks, tactile-only and visual–tactile testing, active-touch standardization, instrumented material characterization, and multisensory or haptic-interface protocols. By comparing these approaches, the review highlights how differences in material selection, sensory access, exploratory movement, subjective rating methods, and physical characterization influence the interpretation of tactile perception data. We also discuss key research gaps, including the need for standardized reporting, cross-material benchmark datasets, diverse participant populations, improved control of touch behavior, and stronger links between tactile perception experiments and material-design guidelines.

## 1 Introduction

Touch is a primary sensory channel through which humans evaluate and interpret the physical properties of materials. Through direct contact with the skin, individuals can perceive surface texture, roughness, softness, hardness, friction, temperature, wetness, stickiness, and compliance. These tactile qualities influence object recognition, product preference, perceived comfort, material quality, usability, and affective response (Bergmann Tiest, 2010; Klatzky et al., 2013; Okamoto et al., 2013). In consumer products, for example, the feel of a material can influence perceived quality, comfort, and user satisfaction. In biomedical and assistive technologies, tactile properties may affect acceptance, accessibility, and long-term usability, particularly for individuals with altered sensory processing. As a result, tactile perception has become an important area of study across materials science, textiles, product design, haptics, human–computer interaction, architecture, rehabilitation engineering, and assistive technology.

However, the perception of materials through touch is a complex multisensory process involving both cutaneous and kinesthetic inputs. Cutaneous receptors in the skin respond to local deformation, vibration, pressure, and thermal exchange, while kinesthetic feedback from muscles and joints provides information about movement, force, and object resistance. This active nature of touch makes tactile perception highly dependent on both the physical properties of the material and the manner in which the material is explored. Therefore, experimental studies of tactile material perception must carefully define not only the materials being tested but also the manner in which participants interact with them. In the last 5 years, experimental research on tactile perception of materials has expanded across several classes of materials, including textiles, synthetic fibers, plastics, wood, building materials, rubbers, textured surfaces, and haptic overlays. Studies differ in the number and type of material samples, the use of tactile-only vs. visual–tactile conditions, the control of exploratory movements, the choice of rating scales, the number of participants, and the physical parameters measured alongside human perception. This methodological diversity reflects the complexity of tactile perception, but it also makes direct comparison across studies difficult. By synthesizing these methodological approaches, this mini-review aims to identify common practices, highlight sources of variability, and provide guidance for designing future experimental studies on tactile perception of materials.

## 2 Typical experimental workflow

We reviewed literature published over the past 5 years to capture the most recent experimental studies and methodological developments. The search was conducted using keywords such as “tactile perception,” “haptics,” and “sensory perception,” and studies were subsequently narrowed based on their relevance to the topic. After applying these criteria, 18 studies were selected for detailed review.

Table 1 summarizes these studies and provides a brief description of the experimental methods employed. From the aforementioned studies, a common experimental workflow emerges. It is the one that most studies followed with only minor variations. As shown in the Figure 1, a typical experimental workflow in tactile perception studies began with the careful selection of material samples. The selected samples represented a meaningful range of tactile properties, such as roughness, smoothness, softness, hardness, friction, warmth, wetness, or stickiness. In recent studies, material sets have included textiles, synthetic fibers, plastics, wood, building materials, rubbers, textured gratings, soft materials, and haptic overlays (Mehta et al., 2024; Lee et al., 2025; Sousa et al., 2022; Kudaligama and Udawattha, 2024). The purpose of this stage was to create a sample set that is diverse enough to produce measurable perceptual differences, while still being controlled enough to allow comparison across materials. For example, if the study focused on roughness perception, the selected samples varied systematically in surface texture or roughness amplitude. If the study focused on softness, the samples varied in stiffness or surface geometry. Careful sample selection was therefore essential because it determined the range of tactile experiences that participants later evaluated.

**Table 1:** Recent experimental studies on tactile perception of materials.

Study	Description
Lee et al. (2025)	Measured roughness and tensile properties of synthetic fibers, then collected human smoothness/roughness ratings using a 5-point tactile scale.
Ikejima et al. (2025)	Combined human sensory and affective adjective ratings with biomimetic tactile-sensor measurements of force, vibration, and temperature across everyday materials.
Guo and Zhang (2025)	Asked participants to touch dry fabrics at different temperatures and rate perceived surface roughness and wetness.
Pasqualotto et al. (2025)	Examined whether touching hard or soft urethane rubbers influenced affective visual processing in a multisensory task.
Zhang et al. (2025)	Compared visual, tactile, and visual–tactile warmth perception of wood surfaces across different wood species.
Devillard et al. (2025)	Recorded haptic, audio, and visual signals during bare-finger exploration of textured surfaces and collected subjective ratings.
Kyriazis et al. (2026)	Combined human finger-friction experiments with acoustic measurements and sensory ratings of fabric feel.
Mehta et al. (2024)	Asked non-expert participants and textile experts to rate textile swatches using descriptors such as hardness, roughness, stickiness, and hairiness.
Roberts et al. (2024)	Used tactile-only and visual–tactile pairwise roughness judgments to test how vision affects tactile texture perception.
Kudaligama and Udawattha (2024)	Used tactile assessment, blindfolded navigation, and real-environment exposure to study perception of building material textures.
Wang et al. (2024)	Compared perceived softness of surfaces with different shapes to examine how surface geometry alters softness perception.
Fan and Zhang (2024)	Compared moist and dry fabrics and tested cooled dry materials to study the roles of temperature, thickness, and friction in wetness illusion.
Nalbantoǧlu et al. (2024)	Used material videos and sound-symbolic words to test whether language-related auditory cues alter perceived softness.
Coe et al. (2023)	Tested tactile detection of virtual straight lines using different overlay materials combined with lateral vibration feedback.
Sousa et al. (2022)	Asked participants to evaluate plastic car-interior textures under tactile-only and visual–tactile conditions using psychophysical and affective descriptors.
Leventini et al. (2022)	Quantified tactile perception of vellum using friction measurements and surface roughness characterization.
Metzger and Toscani (2022)	Studied how vibratory signals produced during tactile exploration support perceptual representations of material properties.
Degraen et al. (2021)	Digitized fabric surface microgeometry, reproduced it using 3D printing, and compared original and replicated textures in a psychophysical experiment.

**Figure 1:**
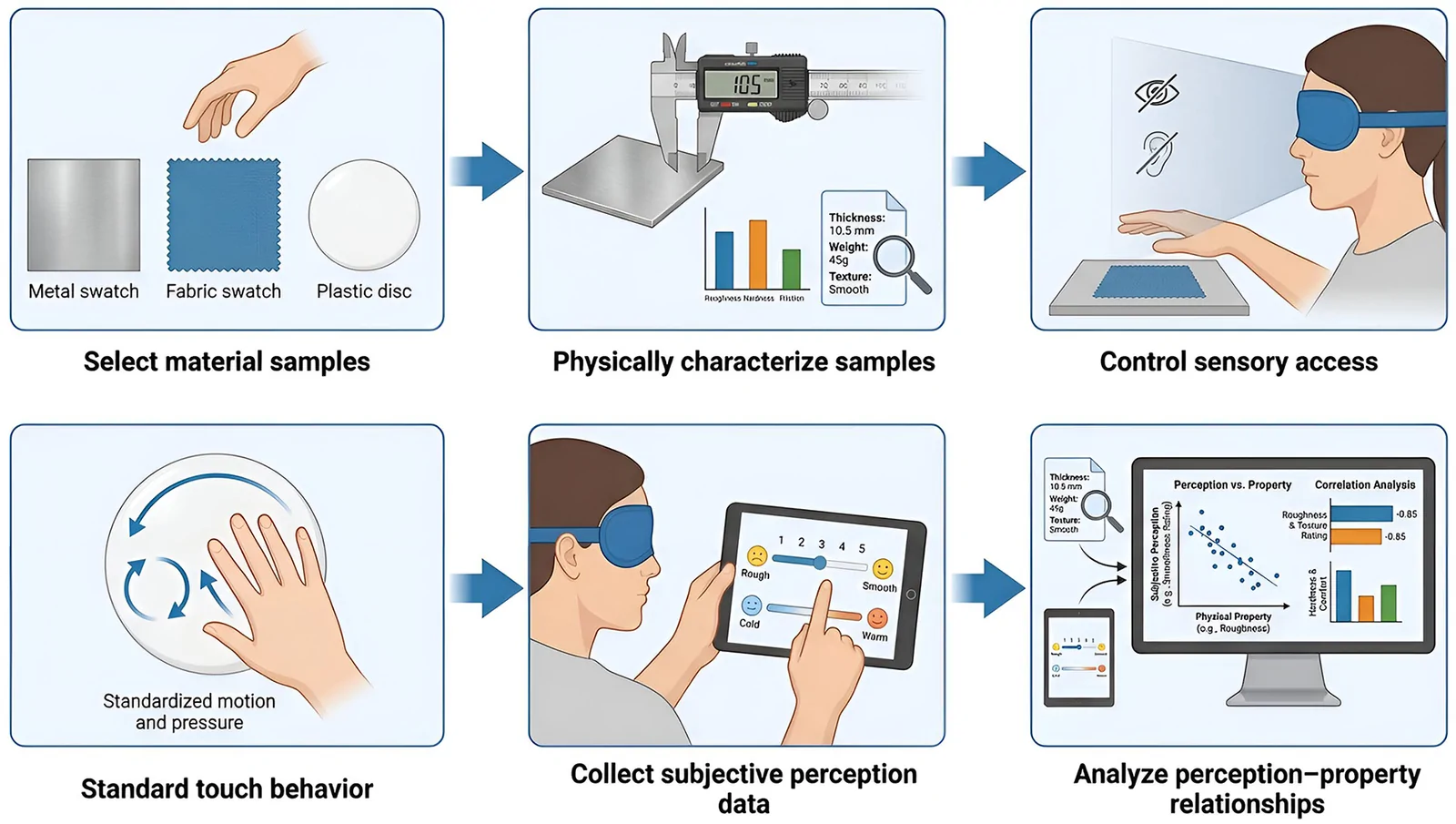
Experimental workflow.

After the materials were selected, the next step was to physically characterize the samples. This stage involved measuring material properties that were expected to influence tactile perception. Depending on the research objective, investigators measured surface roughness, friction coefficient, stiffness, hardness, tensile properties, temperature, thermal properties, acoustic emission, vibration response, or force-displacement behavior. These objective measurements provided an engineering description of each material and allowed researchers to connect subjective perception with measurable physical parameters (Lee et al., 2025; Kyriazis et al., 2026; Devillard et al., 2025). This stage was particularly important because it allowed tactile perception to be interpreted not only as a subjective experience but also as a response to quantifiable material properties.

The third step was to control sensory access during the experiment. Since material perception is often multisensory, researchers decided whether participants will evaluate the samples through touch alone, vision alone, or a combination of vision and touch. In tactile-only protocols, visual information was restricted using blindfolds, curtains, occluding boxes, or hidden sample arrangements. This allowed researchers to isolate tactile perception and reduce the influence of visual cues such as color, gloss, shape, or visible texture. In visual–tactile protocols, participants were allowed to see and touch the materials simultaneously, which more closely resembled real-world interaction. Comparing tactile-only and visual–tactile conditions helped determine how visual information modified judgments of roughness, softness, warmth, comfort, or material quality (Roberts et al., 2024; Sousa et al., 2022; Kudaligama and Udawattha, 2024; Zhang et al., 2025). The underlying explanation for this step is that the same material might be perceived differently depending on whether it is only touched or both seen and touched.

The fourth step was to standardize touch behavior. Tactile perception depends strongly on how a participant interacts with a surface. The same material may feel different when it is tapped, pressed, rubbed, stroked, or explored freely. Therefore, many studies specified the exploratory action used during testing. Participants were instructed to use the dominant index finger, slide the finger in one direction, apply light pressure, press normally, stroke the surface for a fixed duration, or compare two samples using the same motion. Such standardization reduced variability caused by differences in contact force, finger speed, contact area, and exploration time (Lee et al., 2025; Mehta et al., 2024; Devillard et al., 2025). Sliding motion produces skin vibration and frictional cues that strongly contribute to perceived texture. Thus, the choice between standardized and free exploration depended on whether the study prioritized experimental control or ecological validity.

The fifth step was to collect subjective perception data. Once participants interacted with the material samples, their tactile experience was recorded using rating scales, descriptor lists, forced-choice judgments, pairwise comparisons, or detection tasks. Rating protocols commonly ask participants to score attributes such as roughness, smoothness, softness, hardness, stickiness, warmth, wetness, pleasantness, or comfort using Likert-type scales or semantic differential adjective pairs. Pairwise-comparison protocols ask participants to choose which of two samples feels rougher, softer, wetter, warmer, or more pleasant. These methods convert tactile experience into quantitative data that can be analyzed statistically (Mehta et al., 2024; Roberts et al., 2024; Fan and Zhang, 2024). In some studies, affective or usability-related responses such as preference, comfort, perceived quality, or pleasantness were also collected, because tactile perception often influenced how users evaluated materials in real-world applications.

The final step was to analyze perception–property relationships. In this stage, subjective tactile ratings were compared with measured material properties to identify which physical variables explained perception. Common analytical approaches included correlation analysis, regression modeling, analysis of variance, principal component analysis, multidimensional scaling, ordinal logistic regression, and machine-learning methods. The analysis translated experimental observations into design knowledge. By identifying the physical properties that most strongly influence tactile perception, researchers can guide the development of materials, surfaces, haptic interfaces, and assistive technologies that produce desired sensory experiences.

## 3 Limitations

Despite the experimental workflow that we have explained in the previous section to understand the commonality in the protocols, the field remains methodologically fragmented. One of the most important gaps is the lack of a common experimental framework for studying tactile perception of materials. Recent studies have used a wide range of protocols, including Likert-scale ratings, semantic differential scales, forced-choice judgments, tactile-only testing, visual–tactile testing, and instrumented measurements (Mehta et al., 2024; Roberts et al., 2024; Kyriazis et al., 2026; Ikejima et al., 2025). This diversity is useful because tactile perception is a broad and multidimensional phenomenon. However, it also makes comparison across studies difficult. For example, a roughness rating obtained from a five-point scale may not be directly comparable to a roughness judgment obtained from a pairwise discrimination task. Similarly, a tactile-only evaluation may produce different results from a visual–tactile evaluation, even when the same material is tested.

Another limitation is that many studies focus on a single class of materials. This is understandable because testing one material family, such as textiles, synthetic fibers, wood, plastics, or building materials, allows researchers to control specific material variables more carefully (Lee et al., 2025; Mehta et al., 2024; Sousa et al., 2022; Kudaligama and Udawattha, 2024). However, this approach limits the ability to determine whether tactile descriptors such as roughness, softness, warmth, stickiness, or pleasantness are general perceptual dimensions or whether they are strongly dependent on the material category. In real-world interaction, people rarely experience materials in isolation from other material classes. A user may move from touching glass to fabric, wood, plastic, rubber, or metal within the same environment. For this reason, future studies should consider developing benchmark material sets that include multiple material families tested under the same protocol. Such datasets would make it possible to compare tactile perception across materials more systematically and to identify which perceptual dimensions are broadly generalizable. Participant diversity also remains an important area for future research. Many experimental studies rely on healthy adult participants, and in some cases, relatively small participant groups. While this is common in controlled laboratory research, it limits the generalizability of findings to broader populations. Tactile perception may vary with age, sensory sensitivity, professional expertise, cultural experience, neurodevelopmental profile, or tactile impairment. This is particularly relevant for applications in inclusive design, assistive technology, rehabilitation, and human–computer interaction. For example, a material that is perceived as pleasant, smooth, or comfortable by one group may be perceived as irritating, slippery, or overstimulating by another.

A further challenge is the limited control and reporting of touch behavior. Tactile perception is strongly influenced by how a participant explores a material. Sliding, pressing, rubbing, tapping, and stroking generate different mechanical signals at the skin–material interface. Even within the same exploratory action, variables such as sliding speed, normal force, contact area, finger posture, skin hydration, and contact duration can influence perceived roughness, friction, softness, or warmth. Some recent studies have begun to address this issue by standardizing finger use or combining perceptual ratings with frictional, acoustic, or mechanical measurements (Lee et al., 2025; Mehta et al., 2024; Kyriazis et al., 2026; Devillard et al., 2025). Nevertheless, these parameters are not reported consistently across the literature. More detailed reporting of exploratory behavior and contact conditions would improve reproducibility and would make it easier to interpret differences between studies. The relationship between physical material properties and subjective perception also requires further integration. Some studies measure objective properties such as roughness, friction, tensile behavior, vibration, acoustic emission, temperature, or sensor response, while others focus mainly on subjective ratings (Lee et al., 2025; Ikejima et al., 2025; Kyriazis et al., 2026; Devillard et al., 2025). Thus, future studies could work on addressing the limitations discussed in this section.

## 4 Directions for future work

In addition to addressing the limitations of the current experimental protocols, it is also crucial to understand the effects of specific material characteristics on tactile perception. There are some studies that attempt to isolate individual material characteristics that are necessary for establishing fundamental stimulus-perception relationships. For example, Hokari and Takeda (2025) used aluminum-alloy plates with different surface roughness values and found that increasing roughness increased perceived roughness and decreased perceived comfort. By simultaneously measuring finger vibration, the study also connected a defined surface characteristic with the mechanical stimulation generated during touch. Similarly, Yanagibashi and Miki (2022) fabricated tactile samples in which ridge and groove widths were independently controlled, demonstrating that groove width contributed more strongly to perceived roughness and that conventional roughness parameters alone were insufficient to predict perception. Controlled experiments using precisely manufactured gratings have also shown how texture scale, exploratory movement, and contact forces influence roughness discrimination (Roberts et al., 2020), while systematically designed three-dimensional textures have enabled quantitative models relating surface geometry to perceived roughness (Tymms et al., 2018). Such studies minimize confounding between material identity and individual surface characteristics, provide a stronger basis for causal inference, and permit the determination of perceptual thresholds and response functions.

At the same time, there is a need to balance experimental control with ecological validity. Controlled laboratory protocols are valuable because they isolate specific variables and improve repeatability. However, real-world material perception often occurs under more complex conditions, where vision, touch, movement, emotional expectation, repeated handling, and task context interact. Some recent studies cited above have begun to incorporate more naturalistic conditions, such as real-environment exposure, bare-finger interaction, or visual–tactile evaluation. This direction is promising because it connects laboratory findings to everyday material experience. Moreover, studies concerned with product use, comfort, accessibility, or material preference should permit more natural exploratory behaviors and representative environmental conditions because restricting participants to a single movement may exclude strategies normally used to obtain tactile information. For example Aluminum is commonly encountered as part of products such as laptop housings, where perceived touch may also be influenced by surface coatings, edge geometry, device temperature, visual appearance, and the manner in which the laptop is held or operated. Thus, as studied by Hokari and Takeda (2025) aluminum-alloy plates with systematically varied roughness can isolate the influence of surface topography under controlled conditions. Similarly, a standardized plastic disc can isolate the effects of surface texture or hardness, but real-world plastic surfaces, such as appliance controls, electronic-device components, packaging, or automotive interiors, are experienced in combination with shape, curvature, temperature, visual cues, and functional context.

Based on the discussion above, a tiered protocol can be adopted. The standardized core could include common reporting requirements, benchmark materials, randomized sample presentation, objective material characterization, and measurements of contact force, movement speed, contact duration, and environmental conditions. The application-specific module could then incorporate freely chosen movements, visual-tactile access, task-relevant materials, representative users, and realistic environmental conditions. Whenever feasible, the same participants and materials could first be evaluated under standardized conditions and subsequently during a naturalistic task. Findings that remain consistent across both conditions may be considered more robust and generalizable, whereas differences can reveal how product geometry, context, and exploratory behavior modify tactile perception. Thus, controlled and ecologically valid protocols should be treated as complementary stages: the former establishes fundamental relationships and facilitates cross-study comparison, while the latter determines whether those relationships generalize to everyday material interactions.

## 5 Conclusion

Future work in tactile-perception research will benefit from more systematic reporting of experimental conditions, including participant instructions, sensory access, exploratory movements, contact duration, rating-scale design, sample order, and the physical characterization methods used. Greater attention to participant diversity is also essential, particularly in understanding how protocols may need to be adapted for individuals with different sensory-processing profiles. Finally, a more integrated approach that considers how tactile cues interact with visual, auditory, and other non-tactile information will help clarify how people form judgments of comfort, quality, pleasantness, and usability. Together, these improvements can support more robust, comparable, and ecologically valid research on how humans perceive and evaluate materials.
